# Impaired Processing in the Primary Auditory Cortex of an Animal Model of Autism

**DOI:** 10.3389/fnsys.2015.00158

**Published:** 2015-11-16

**Authors:** Renata Figueiredo Anomal, Etienne de Villers-Sidani, Juliana Alves Brandão, Rebecca Diniz, Marcos R. Costa, Rodrigo N. Romcy-Pereira

**Affiliations:** ^1^Brain Institute, Federal University of Rio Grande do NorteNatal, Brazil; ^2^Montreal Neurological Institute, McGill UniversityMontreal, QC, Canada

**Keywords:** animal model of mental disorders, autism spectrum disorders (ASD), auditory perception, inhibitory neurons, cortical mapping

## Abstract

Autism is a neurodevelopmental disorder clinically characterized by deficits in communication, lack of social interaction and repetitive behaviors with restricted interests. A number of studies have reported that sensory perception abnormalities are common in autistic individuals and might contribute to the complex behavioral symptoms of the disorder. In this context, hearing incongruence is particularly prevalent. Considering that some of this abnormal processing might stem from the unbalance of inhibitory and excitatory drives in brain circuitries, we used an animal model of autism induced by valproic acid (VPA) during pregnancy in order to investigate the tonotopic organization of the primary auditory cortex (AI) and its local inhibitory circuitry. Our results show that VPA rats have distorted primary auditory maps with over-representation of high frequencies, broadly tuned receptive fields and higher sound intensity thresholds as compared to controls. However, we did not detect differences in the number of parvalbumin-positive interneurons in AI of VPA and control rats. Altogether our findings show that neurophysiological impairments of hearing perception in this autism model occur independently of alterations in the number of parvalbumin-expressing interneurons. These data support the notion that fine circuit alterations, rather than gross cellular modification, could lead to neurophysiological changes in the autistic brain.

## Introduction

Autism is a neurodevelopmental disorder that affects approximately 1 in 88 children and produces a wide range of sensory, motor and integrative behavioral deficits (Leekman et al., 2007). Clinically, autistic children can show severe intellectual disability with seizures to intense social aversion and frequent stereotypies or, in some cases, a less incapacitating profile of mild social retraction with typical-to-high intellectual performance (Lai et al., 2014). Such heterogeneity of profiles is partly due to the existence of multiple etiological factors underlying the autism spectrum disorder (ASD) associated to the current imprecise diagnostic methods that rely basically on behavioral evaluations (Kapur et al., 2012). In this context, the development of better diagnostic methods and treatments for subtype-specific forms of autism can benefit from studies aimed to characterize reliable electrophysiological autistic endophenotypes (i.e., physiological or biochemical processes altered in the disorder).

It is well described in the literature that hearing abnormalities including hyper/hypo-sensitivity, deficits in acuity and incongruence of auditory perception (i.e., distortions) are frequently observed among autistic individuals (Rosenhall et al., 1999; Davis et al., 2006; Tharpe et al., 2006; Gomes et al., 2008; Madsen et al., 2014). Recent studies also support the idea that sensory dysfunctions in autism may be related to an inhibitory-excitatory imbalance derived from alterations of critical period time course (Rubenstein and Merzenich, 2003; Le Blanc and Fagiolini, 2011). In rodents, genetic and pharmacological models of autism represent an important tool for the characterization of endophenotypes associated with autism and for testing strategies of behavioral rescue.

The valproic acid (VPA) model of autism translates to the animal the prenatal exposure of embryos to the antiepileptic drug VPA, which is shown to significantly increase the odds of autistic births in humans (Christianson et al., 1994; Rodier et al., 1997; Williams et al., 2001; Miyazaki et al., 2005; Christensen et al., 2013). In animals exposed to VPA *in utero*, several autistic-like behaviors tend to appear including reduced social interaction, reduced sensitivity to pain, increased sensitivity to tactile stimuli, diminished acoustic prepulse inhibition, memory impairment/improvement, prolonged repetitive behaviors, altered anxiety and fear behaviors and hyperactivity (Schneider and Przewlocki, 2005; Markram et al., 2008; Bambini-Junior et al., 2011; Brandão and Romcy-Pereira, Unpublished; Edalatmanesh et al., 2013; Kataoka et al., 2013; Kim and Bao, 2013). Electrophysiologically, it was also shown that rats prenatally exposed to VPA *in utero* display changes in the N-methyl-D-aspartate receptor (NMDA)-mediated currents and synaptic plasticity of cortical neurons. Neonatal and adolescent VPA rats show reduced excitability and increased cortical plasticity, whereas adult rats have lower NMDA-mediated currents and reduced cortical LTP (Rinaldi et al., 2007; Silva et al., 2009; Walcott et al., 2011; Martin and Manzoni, 2014). Pups seem to have a higher degree of neuronal connectivity between cortical neurons (Rinaldi et al., 2008). At the cellular level, VPA rats can show reduced number of parvalbumin inhibitory neurons in the somatosensory cortex, impoverished cortical dendritic arborization and reduced dendritic spine distribution (Gogolla et al., 2009; Mychasiuk et al., 2012).

Parvalbumin-positive cells comprise 40% of all GABAergic inhibitory neurons in the mouse primary auditory cortex (AI; Xu et al., 2010). During development, these cells play pivotal roles in shaping receptive fields in different primary sensory areas (del Rio et al., 1994; Huang et al., 1999; Sugiyama et al., 2008), and changes in the distribution of PV cells have been observed in the AI of rats with altered tonotopic maps (de Villers-Sidani et al., 2008). PV cells in the AI are fast-spiking interneurons that receive thalamic inputs and initiate a feedforward inhibition on their target pyramidal cells (Mallet et al., 2005; Galtrey and Fawcett, 2007), enabling upper layers II/III excitatory neurons to refine auditory representation (Li et al., 2014). Thus, appropriate control of numbers and distribution of PV-cells is pivotal for both refinement and maintenance of the receptive fields of excitatory neurons in AI.

In the present study, we investigated the tonotopic organization of the auditory sensory map and the characteristic responses of AI cortical neurons of rats exposed to VPA *in utero*. We also quantified the density and distribution of parvalbumin-positive inhibitory neurons in AI of VPA and control animals.

## Materials and Methods

All procedures were approved by the Ethics Committee for Animal Experimentation of the Federal University of Rio Grande do Norte (UFRN; #044/2011) and by the Animal Care Committee of Montreal Neurological Institute and complied with guidelines of the Canadian Council on Animal Care. In total, 30 Wistar rats (male and female) were used for electrophysiology experiments (*N* = 14) or histological analyses (*N* = 16). VPA (*N* = 8; 6 males and 2 females) and control (*N* = 6; 4 males and 2 females) rats were assigned to electrophysiological recordings at postnatal age 30 (P30) to P48.

### Valproic Acid Administration

To produce VPA rats, two pregnant females with controlled oestrus cycle at embryonic day 12.5 (E12.5) were injected with a solution of VPA (500 mg/Kg; dissolved in NaCl 0.15 M i.p.; Gogolla et al., 2009). E12.5 corresponds to the time of neural tube closure in rats (Rodier et al., 1996). Two control female rats received an injection with the same volume of vehicle (NaCl 0.15 M i.p.) solution and were mated. Pregnant females were maintained undisturbed in the animal care facility and were monitored until delivery—delivery day was considered postnatal day 0 (P0). We did not observed deaths associated to delivery or cannibalism in neonatal offspring. All recorded rats in the VPA group had a kink in the tail by the time of surgery, a common malformation due to embryonic exposure to VPA (Gogolla et al., 2009; Brandão and Romcy-Pereira, Unpublished). Pups were weaned on P21 and housed individually until they reached the age (P30–P48) for auditory mapping or perfusion for immunohistochemistry.

### Auditory Cortical Mapping

**Figure 1:**
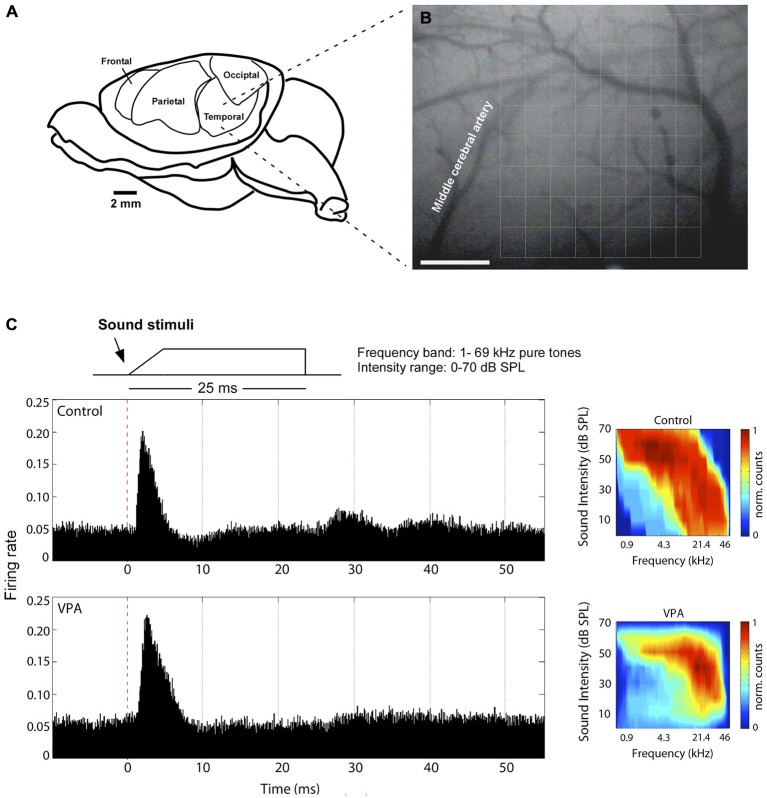
**Electrophysiological mapping of the primary auditory cortex. (A)** Anatomical disposition of the rat’s temporal cortex at left hemisphere. **(B)** Primary auditory cortex is dorsal to rhinal fissure and caudal to middle cerebral artery, where the center of an 8 × 8 microelectrode array (375 × 500 mm grid) was placed for the electrophysiological recordings. **(C)** *Left*, Illustrative neuronal responses in AI to pure tone pulse stimuli. Here, we show one recording site of a control and a valproic acid (VPA) animal. *Right*, Normalized activation plots corresponding to AI on the left. Each value in the map represents the average of three trials for each bin (1 ms). Colors represent interpolated and normalized firing rate counts. Calibration bar = 1 mm.

The left primary auditory cortex of rats was mapped as previously described (de Villers-Sidani et al., 2007; Anomal et al., 2013). Rats assigned to control and VPA group were obtained in equal number from two pregnant females. Briefly, they were anesthetized with a cocktail of ketamine (50 mg/kg, i.p.), xylazine (5 mg/kg, i.p.), acepromazine (1 mg/kg, i.p.), and received an injection of the anti-inflammatory dexamethasone (0.2 mg/kg, i.p.) before surgery. Body temperature was maintained with a heating pad at 36 ± 1^°^C. The cistern magna was drained of cerebrospinal fluid to minimize cerebral edema. The skull was kept fixed by a head holder, leaving the ears unobstructed. The left or right temporalis muscle was reflected exposing the auditory cortex and the dura-mater was resected to broadly uncover AI. AI was anatomically identified as outlined by the medial cerebral artery, dorsal to the rhinal fissure (Figures 1A,B; see also Krubitzer et al., 2011).

Multi-unit electrophysiological recordings were obtained using an array of 64 tungsten microelectrodes (8 × 8 electrodes). In the array, the distance between electrode columns was 375 μm, and between rows 500 μm (Figures 1A,B). The array size was sufficient to encompass the whole cortical area corresponding to AI (3 × 4 mm). Microelectrodes were lowered perpendicular to the cortical surface to depths of 470–600 μm, targeting cortical layers IV and V. The preferential recording depth was 500 μm from pial surface, where spontaneous neuronal activity is characteristic of a thalamo-cortical recipient layer. In all experiments, at least two penetrations of the microelectrode array were done in temporal cortex of each individual rat. In each array penetration, the rat’s auditory system was stimulated by acoustic waves generated by TDT System III (Tucker-Davis Technologies, Inc., Alachua, FL, USA), through speakers that delivered sound to the contralateral ear in an open field mode. Frequency-intensity receptive fields were reconstructed by presenting pure tones of 66 frequencies (1–70 kHz, 0.1-octave increments, 25-ms duration, 5-ms ramps) at eight sound intensities (0–70 dB SPL in 10-dB increments), in a rate of 2 stimuli per second. Neuronal responses were amplified (10,000×), filtered (0.3–3 kHz) and monitored online while recording (sampling rate of 20 kHz). We used the software package SigGen and OpenEx (Tucker-Davis Technology, Inc., Alachua, FL, USA) to generate acoustic stimuli, monitor cortical response properties online and store data for off-line analysis. Evoked spikes from multiple neurons were collected at each recording site of the array to reconstruct receptive fields.

### Data Analysis

**Figure 2:**
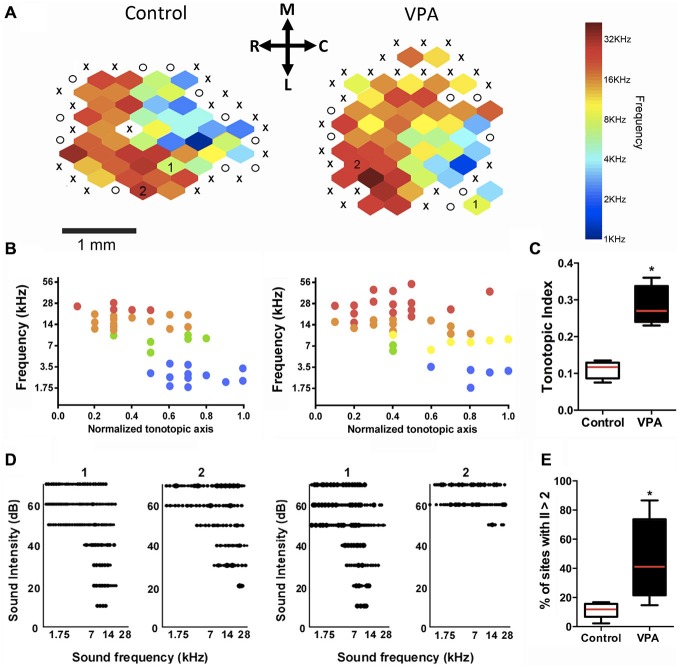
**Embryonic administration of VPA alters the tonotopic organization and receptive fields of AI. (A)** Tonotopic maps of a control (*left*) and VPA rat (*right*). *X marks* = lack of tone-evoked responses; *O marks* = sites outside AI. Color bar represents the spectral distribution of sound frequencies that elicited neuronal responses in AI. **(B)** Distribution of characteristic frequency (CF) responses plotted against the normalized tonotopic axis from representative maps shown in **(A)**. **(C)** VPA animals show higher tonotopic index compared to controls, indicating a disorganization in their primary auditory cortical maps (^*^p < 0.01, Student’s *t*-test). **(D)** Representative receptive fields from two sites in the control and VPA maps at two different characteristic frequencies (see corresponding numbers inside each map). Note that the receptive field of VPA neuron #2 has a distinct flat peak shape and a high-threshold response for sound intensity, which contrasts to v-shaped receptive fields of AI neurons in control animals. **(E)** VPA animals show more sites with irregularity index (II) higher than 2, indicating a reduced relative number of v-shaped tuning curves (^*^p < 0.05, Students *t*-test). Calibration bar = 1 mm.

We initially identified the characteristic frequency (CF; frequency at which neurons respond at lowest intensity threshold) and receptive field border of each cortical site. CFs were automatically defined as the tip of the tuning curve and tonotopic cortical maps were generated by Voronoi tessellation using custom-made MatLab routines. The center of each polygon in the map corresponds to the site of one microelectrode penetration and the colors represent the CF associated with neurons located in that site. Polygon area is proportional to the distance between neighboring penetrations. AI border was determined by the characteristic responses of the recorded neurons and by the topographic organization of represented frequencies in the cortex. Boundaries of the primary auditory cortex were functionally determined using the following criteria: (1) primary auditory neurons generally have a continuous, single-peak shape in the tuning curve (intensity-frequency plot; Figure 1C) and (2) CF of AI neurons are tonotopically organized with high frequencies represented rostrally and low frequencies represented caudally (Figure 2A). Moreover, (3) recording sites in AI typically presented strong responses evoked by low intensity tones (Figure 1C). To distinguish from posterior auditory field (PAF) and ventral auditory field (VAF), which are contiguous to AI, we considered their distinct tuning curves properties, as well as their particular topographical organization of responses to sounds of different frequencies (Polley et al., 2007). PAF was distinguished as a narrow band of cortex just caudal to AI, containing few and broad receptive fields, which exhibited discontinuous responses to sound frequencies. VAF, in the posterior ventral boundary, was identified by its patchy profile of responses to frequencies at low-threshold intensities (<30 dB SPL) but with little or no tone-evoked responses above this level. Recording sites outside AI were responsive only to higher intensity sounds, or were not reliably excited by tonal stimuli (Bao et al., 2003).

**Figure 3:**
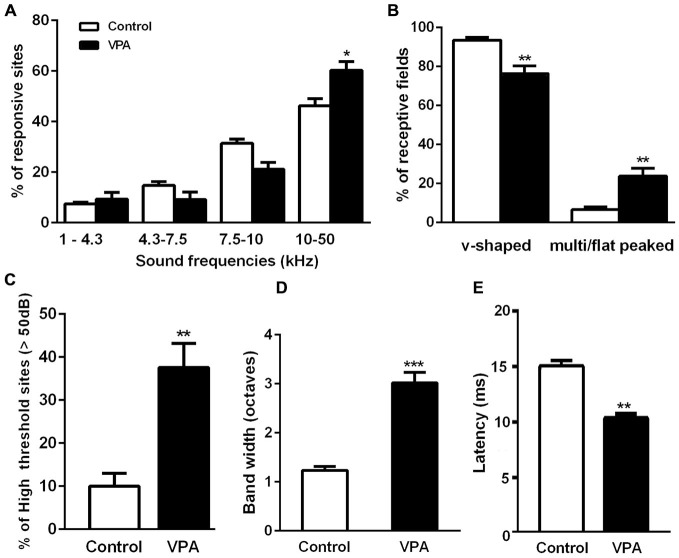
**Embryonic administration of VPA alters the maturation of receptive fields in AI. (A)** Fraction of AI responsive sites at distinct sound frequency bands. VPA rats present larger cortical representation for sound frequencies above 10 kHz (*F*_(3, 30)_ = 63.37, *p* < 0.05; two-way ANOVA with repeated measures, Bonferroni’s multiple comparison test). **(B)** Relative number of single and multi/flat-peaked receptive fields in each group. V-shaped receptive fields are significantly reduced in VPA animals as compared to controls, whereas multi-peaked sites are increased in this group (*p* < 0.01, Student’s *t*-test). **(C)** Sound intensity threshold of AI neurons in VPA rats is significantly higher than those measured in control animals (*p* < 0.01, Student’s *t*-test). **(D)** Frequency response bandwidth 10 dB above sound intensity threshold (BW10). Note that BW10 is significantly augmented in VPA rats (*p* < 0.0001, Student’s *t*-test). **(E)** Latency of neuronal responses in AI was significantly decreased in VPA rats as compared to controls (*p* < 0.01, Student’s *t*-test). Values are means ± SEM. ^*^p < 0.05; ^**^p < 0.01; ^***^p < 0.0001.

Tuning curves were classified as v-shaped and multi-peaked according to previous studies in AI (Bao et al., 2004; Zhou and Merzenich, 2007; Anomal et al., 2013). The receptive field irregularity index was used to quantify eventual differences between control and experimental animals. The irregularity index was defined as [Corr(0, 0) − (Corr(1, 0) + Corr(0, 1))/2]/Corr(0, 0)1/2 minus a constant number of 3, where Corr(0, 0) represents the central term of the receptive field and Corr(0, 0) − (Corr(1, 0) + Corr(0, 1))/2 represents the periphery of the receptive field (Bao et al., 2003). After that, we calculated the percentage of sites presenting irregularity index above 2. Higher irregularity index means that the tuning curve is less v-shaped. Single-peaked sites were identified as a well-defined v-shaped tuning curve, containing one apex or one CF. Tuning curves without apex (flat design) or containing more than one apex were seen as flat/multi-peaked sites. For flat-peaked tuning curves, the median frequency at minimal intensity was chosen as the CF. For tuning curves presenting multiple-peaks, the CF was defined at the apex with lowest threshold. BW10 was defined as the range of frequencies in octaves (frequency response range) that was able to elicit neuronal responses 10 dB above intensity threshold. Characteristic frequencies, intensity thresholds and BW10 were computed using custom-made Matlab routines (MathWorks, Natick, MA, USA). Percentages of sites described in our results were obtained from the total number of recorded sites inside AI (see Figure 3). Response latency was defined as the time from stimulus onset to the multiunit response level 4 standard deviations above the mean pre-stimulus firing rate level.

In order to compare AI maps, the tonotopic axis was normalized in the rostro-caudal axis within a 0.0–1.0 range. The normalized tonotopic axis was calculated by rotating the map to make horizontal a linear function fit of the penetration coordinates using a least squares method. After rotation, penetration coordinates were vertically collapsed on and normalized to a 0–1 range. A tonotopic index was calculated as the average minimum distance from each coordinate on the scatterplot of AI normalized axis to the line describing the perfect tonotopic axis, connecting (0, 0) and (1, 1) after converting the logarithmic frequency range (1–63 kHz) to a linear range (0–1; Zhang et al., 2001). It can be interpreted as a measure of the imprecision in tonotopicity of each map, in which a higher value reflects a less organized tonotopic gradient. Recorded sites were plotted according to their position in normalized coordinates vs. CFs.

### Brain Sectioning and Immunofluorescence Processing

In total, 43 brain sections from 16 rats were used for histological analysis aging from P35–P40 (Control, *N* = 9; VPA, *N* = 7 animals). Animals were anesthetized with sodium thiopental (80 mg/kg, i.p) and transcardially perfused with 100 mM saline phosphate-buffered solution (PBS pH 7.4 followed by 4% paraformaldehyde diluted in 100 mM phosphate-buffered solution, PB pH 7.4). Once brains were dissected, they were post-fixed overnight in the same fixative solution at 4^°^C and cryoprotected in sucrose 30% dissolved in PB. The brains were frozen at −40^°^C in dry-ice cooled isopentane. Brain sections (20 μm) were obtained in a cryostat (Microm, HM 550), mounted on glass slides and stored at −80^°^C.

For immunohistochemistry, sections were washed in 10 mM PBS for 10 min, permeabilized and blocked for 2 h in blocking buffer (5% normal goat serum/0.5% triton X-100 in 10 mM PBS) at room temperature. Sections were incubated in primary antibody solution (rabbit anti-parvalbumin at 1:1000; Swant PV25) diluted in blocking buffer overnight, 4^°^C. Sections were then washed three times for 10 min in PBS, and incubated with secondary antibody for 2 h at room temperature in the dark (AlexaFluor 546, goat anti-rabbit at 1:1000, Invitrogen, AI1010). After incubation, sections were washed in PBS, three times for 10 min.

Cortical histology including layer organization could be identified using DAPI nucleic acid stain. DAPI working solution was prepared by diluting DAPI stock solution (1:1000 in PBS with Triton X-100 0.5%; Sigma, D9542) and using 150 μl on each slide containing brain coronal sections. Sections were incubated with DAPI for 5 min, washed in PBS and mounted with anti-fading Fluoromount (Polyscience, 18606). Slides were stored at 4^°^C for until microscopic quantification.

Parvalbumin-positive neurons (PV neurons) were blindly counted in sections from rostral to caudal AI encompassing 1.5 mm in the antero-posterior axis (from Bregma AP, −3.3 to −4.8 mm). AI was identified as Au1, according to Paxinos and Watson (2007). All cortical layers were clearly defined in AI through a DAPI filter. Layer IV was identified as a thick and well-delimited band of cells, stacked in high density. The adjacent AuD (dorsal auditory field) and AuV (VAF) were avoided. The distinction of cortical layers in these regions is much less clear than in AI. For the AI border, cells outside AI are spread, and layer IV cell density is clearly reduced. Therefore, the dorsal and ventral borders of AI were defined using layer IV as a reference.

Primary auditory cortex, neocortical layers and parvalbumin cells were visually inspected using a Zeiss Imager M.2 ApoTome.2 microscope equipped with epifluorescence and a *x*-*y*-*z* stage encoding system attached to a computer (MBF Bioscience, Stereo-Investigator).

### Statistical Analysis

Data are presented as mean ± SEM. Statistical significance was evaluated using either Student’s *t*-test or two-way ANOVA with repeated measures with Bonferroni’s multiple comparison test. The significance level was set as *p* < 0.05.

## Results

Multiunit neural recordings were obtained from 1600 electrode positions consisting of 832 sites in VPA animals (*N* = 8) and 768 sites in control animals (*N* = 6). Each animal underwent two recording sessions with a 64-channel electrode array, totalizing 128 recording sites per animal. Because the area of each electrode array extended beyond AI, encompassing AI and surrounding auditory cortical regions, we reconstructed AI maps using a subset of 287 responsive sites in the control group and 256 sites in the VPA group (Figure 2A).

The comparison of cortical maps was done by the computation of a tonotopic index, which was used as a metric to quantify how close is the spatial distribution of frequency responses in a map compared to the ideal theoretical smooth tonotopy along the antero-posterior axis of AI (Figures 2B,C). Our results showed that AI maps obtained from VPA rats had significantly higher tonotopic index than controls, indicating a more disorganized map in rats prenatally exposed to VPA (Figure 2C; VPA = 0.28 ± 0.03, Controls = 0.11 ± 0.01; Student’s *t*-test, *p* = 0.0004). In addition, receptive fields in VPA animals showed to be less tuned to its characteristic frequencies than those in controls (Figure 2D). The percentage of sites with irregularity index above two was greater in the VPA group (mean ± SEM; 41 ± 10%) than in controls (11 ± 2.2%; Student’s *t*-test, *p* = 0.025; Figure 2E), meaning that the primary auditory cortex of VPA rats has more irregularly shaped receptive fields than controls.

We also observed that AI maps of VPA-treated animals showed an overrepresentation of higher sound frequencies as compared to controls. In fact, the percentage of sites responsive to high frequencies (10–50 kHz) was significantly larger in VPA than in controls (Figure 3A; VPA = 60.29 ± 3.42% of sites, Controls = 46.28 ± 2.81% of sites; *F*_(3, 30)_ = 63.37, *p* < 0.0001; two-way ANOVA with repeated measures followed by Bonferroni’s multiple comparison test, *t*_(40)_ = 3.16, *p* < 0.05). Although statistically not significant, we could notice a decrease in the number of receptive fields responding to frequencies in the range 4.3–10 kHz (Figure 3A). Control animals displayed a typical tonotopic organization of AI with sound frequencies (1–40 kHz) represented in bands of frequencies following a caudal-to-rostral organization (Figure 2A). Higher sound frequencies were rostrally represented in AI, while lower frequencies were progressively represented in caudal areas (Figure 2B).

In control animals, we observed a predominance of typical single-peaked and v-shaped receptive fields in AI (Figure 3B). VPA animals, in contrast, showed reduced number of AI sites with v-shaped tuning curves compared to controls. In these animals, there was a significant enhancement in the fraction of recorded sites with multi and flat-peaked shape (Figure 3B; *v-shaped sites*: VPA = 76.25 ± 3.98% of sites, Controls = 93.47 ± 1.39% of sites; *multi/flat peaked sites*: VPA = 23.75 ± 3.98% of sites, Controls = 6.53 ± 1.39% of sites; Student’s *t*-test, *t*_(12)_ = 3.59, *p* = 0.0037 of each comparison). Moreover, VPA animals displayed increased number of high-intensity threshold sites in AI with onset responsiveness to sound intensities above 50 dB (Figure 3C; VPA = 37.58 ± 5.62% of sites, Controls = 9.99 ± 3.01% of sites; Student’s *t*-test, *p* = 0.002).

We also measured receptive field width by quantifying the range of characteristic frequencies 10 dB above the receptive field’s intensity threshold (BW10). Significantly larger BW10 were detected in AI of VPA rats as compared to controls (Figure 3D; VPA = 3.02 ± 0.21 octaves, Controls = 1.24 ± 0.08 octaves; Student’s *t*-test, *p* < 0.0001). Noteworthy, the latency of neuronal responses to sound stimuli in VPA animals was significantly decreased in comparison to controls (Figure 3E; VPA = 10.27 ± 0.51 ms, Controls = 14.97 ± 0.67 ms; Student’s *t*-test, *p* = 0.0049). Correlation between receptive field parameters is shown in Supplementary Figure S1.

### Parvalbumin-Expressing (PV) Inhibitory Neurons in the Primary Auditory Cortex

**Figure 4:**
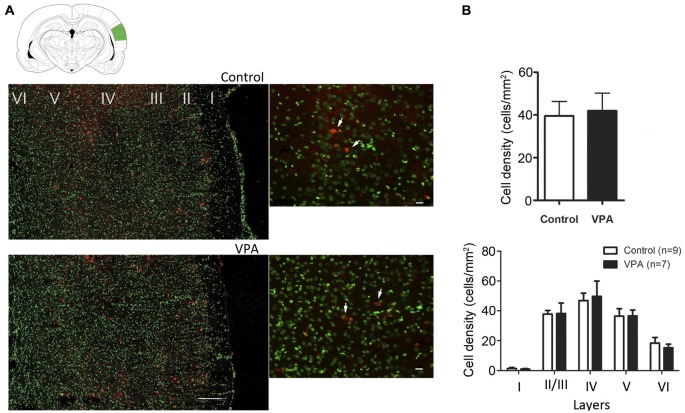
**Quantitative distribution of parvalbumin-positive neurons in AI of VPA and control animals. (A)** Cell density of parvalbumin-positive neurons in VPA animals is not significantly different from controls (*p* = 0.3251, Student’s *t*-test). **(B)** No alteration on cell densities were found in AI of rat models of autism (*p* = 0.8296, Student’s *t*-test).

It has been suggested that changes in the number of PV inhibitory neurons during development and adulthood contribute to some of the functional deficits observed in ASD (Gogolla et al., 2009). However, in the VPA and several genetic models of ASD, divergent results for number and distribution of PV inhibitory neurons have been observed for different brain areas (Gogolla et al., 2009). To test whether the electrophysiological changes observed in VPA-treated animals could be explained by a difference in the numbers, density or layer distribution of PV-cells in AI, we quantified these variables in AI of VPA (*N* = 7) and control (*N* = 9) animals at P35–40. We observed that the total number of PV neurons in AI was similar between VPA-treated and control rats (VPA = 50.90 ± 7.55 cells, Controls = 62.72 ± 8.34 cells, Student’s *t*-test, *p* = 0.3251). Next, we calculated the density (number of cells/mm^2^) of PV inhibitory neurons in the whole AI or within individual cortical layers (Figure 4). We observed that neither the density of these cells per area (Figure 4A; Student’s *t-test*, *p* = 0.3251) nor the layer density (Figure 4B; Student’s *t-test*, *p* = 0.8296) was affected in VPA animals, as compared to controls. Thus, our histological analysis does not support the notion that electrophysiological alteration in AI would be caused by changes in the total number of PV-expressing inhibitory neurons. Besides, there was no significant difference in the overall density of PV-positive cells in AI (Supplementary Figure S2; two-way ANOVA *F*_(1, 12)_ = 0.45, *p* > 0.05; treatment (VPA-Control) *vs.* sex (Male-Female) or in cell density per layer (two-way ANOVA, treatment effect, *p* > 0.05) between male and female of either control and VPA rats.

## Discussion

Hearing disorders are common in autistic children. According to Klin (1993) about 33–46% of autistic individuals show some form of auditory deficit. A series of studies have also reported that altered brainstem and cognitive auditory evoked potentials (Rosenhall et al., 1999; Kulesza et al., 2011), morphological abnormalities in the auditory system (Rodier et al., 1997), abnormal lateralization during language processing and GABAergic and serotonergic neurochemical dysfunctions are associated to abnormal auditory processing in autism (Hitoglou et al., 2010). In the present study, we used a rodent model of autism, generated by prenatally exposing rats to the anti-epileptic and epigenetic modulator VPA, in order to investigate the tone-evoked properties and spatial organization of auditory receptive field responses across the primary auditory cortex. In order to assess the cellular organization of the cortical inhibitory circuitry, an important element for shaping and refining auditory receptive fields, we also quantified the layer distribution of parvalbumin-positive GABAergic interneurons in AI of VPA-treated and control animals.

Our main findings show that rats prenatally exposed to VPA have a disorganized primary auditory tonotopic map with over-representation of high frequencies and abnormal neuronal responses to sound as compared to controls. VPA animals show broadly tuned receptive fields, higher intensity thresholds and shorter onset latencies for neuronal firing following sound presentation. Surprisingly, such atypical tonotopic organization and receptive field responses are neither associated with changes in the total number nor with distinct layer distribution of cortical parvalbuminergic interneurons.

### Primary Auditory Processing in the VPA Model of Autism

In rats, cortical representation of sounds takes place in five auditory cortical fields: primary auditory cortex (AI), anterior auditory field (AAF), posterior auditory field (PAF), ventral auditory field (VAF) and suprarhinal auditory field (SRAF; Polley et al., 2007). In AI, receptive fields are tonotopically organized with responses distributed in a rostro-caudal gradient from low (~1 kHz) to high (~60 kHz) frequencies, with neurons firing at short latency (6–20 ms) after stimulus onset and sharply tuned (v-shaped tuning curves) to a particular CF (Horikawa et al., 1988; Sally and Kelly, 1988; Kilgard and Merzenich, 1999; Doron et al., 2002). Consistent with previous studies, the primary auditory cortex of our control animals was identified as an electrophysiologically well-defined region, in which tonotopically organized bands of responses to low and high frequencies were spatially disposed from rostral to caudal sites. Receptive fields in AI showed a prevalence of v-shaped responses with latency to neuronal firing within ~15 ms of sound presentation and low-intensity thresholds. These responses were compatible with those obtained for animals of similar age (P30–P48; de Villers-Sidani et al., 2007).

In contrast, we observed that sound representation in AI is not properly established in pre-puberal VPA rats. VPA animals showed a significantly less organized gradient of characteristic frequencies across the antero-posterior cortical axis as compared to controls. This was verified by the significantly higher tonotopic index calculated for maps from VPA rats compared to controls. Besides, the primary auditory cortex of VPA animals displayed an over-representation of high frequencies denoted by a significantly larger proportion (~60%) of receptive fields with characteristic frequencies between 10–50 kHz, which concentrated the upper three octaves of the full seven-octave frequency range. It was accompanied by a concurrent decrease, though statistically not significant, in the number of receptive fields responding to frequencies of 4.3–10 kHz. VPA animals also showed less spectral selectivity revealed by broader receptive fields, a feature more often found in low-mid frequency responsive sites. In addition, we also showed that VPA rats have higher thresholds for sound intensity responses compared to controls, suggesting a hearing impairment that consists of a much larger area of AI field that starts responding (increased neuronal firing) at intensities of 50 dB.

In a recent study, Engineer et al. (2014a) also showed that rats prenatally exposed to VPA have deficits in auditory cortical processing. Using speech sound and pure tone stimulations the authors found significant impairments in the processing of spectral and temporal features of the stimulus involving both AAF and AI. Although abnormal cortical responses were more conspicuous in AAF, significant alterations in AI were described such as increased local field responses and reduced peak firing rate to speech sounds. In response to pure tones, it was found that AI receptive fields had increased threshold intensity for neuronal firing and reduced spectral selectivity (higher BW40). These findings represent similar changes as those observed in our study. However, they did not find significant alterations in latency, frequency tuning and tonotopic organization as the ones we have shown. It is possible that the age of AI mapping (P23 as opposed to P30–P48 here) might have contributed to the differences observed in both studies, since there is still significant receptive field plasticity occurring at earlier ages in AI (de Villers-Sidani et al., 2007). This could mask subtle changes in tone-evoked responses that would pass undetectable, only appearing later in time (after P30). It is less likely that the use of a lower VPA dose (500 mg/kg in our study instead of 600 mg/kg in theirs) might have produced a more severe phenotype. Besides, the use of pre-puberal male and female rats - 1/2 (female:male ratio) in the control group and 1/3 in VPA group in the present study does not seem to have cause such effects, since differences in auditory processing between male and females have only been reported in post-puberal females with active estrous cycle and aging (Calas, 2013). Interestingly, in the BXD29-Tlr4^lps-2J^/J mouse model of neurodevelopmental disorder, male and female animals display similar auditory processing impairments (Truong et al., 2013).

Reports on other rodent models of autism were also recently published showing additional abnormalities in sound representation in AI. In the Fmr1 KO mouse model of fragile X syndrome, similar to our findings, it was shown broader AI tuning curves with higher latency variability and an over-representation of high frequencies between 11–25 kHz. Interestingly, these animals also display, altered GABAergic signaling associated to an acoustic hypersensitivity and propensity for audiogenic seizures (D’Hulst et al., 2006; Rotschafer and Razak, 2013). In Fmr1 KO rats, on the other hand, it was shown reduced local field and firing responses associated with reduced responsivity to sounds above 60 dB (Engineer et al., 2014b). In the MeCP2 KO rat model of Rett syndrome, speech sound stimulation also revealed hyperexcitability of AI and temporal processing impairments (Engineer et al., 2015).

It is possible that the neurophysiological abnormalities observed in AI of VPA animals disclose a maturational dysfunction resulting from the VPA interference during the early stages of brain development (E12.5). It has been described that neurons with two or more characteristic frequencies are typical of the immature AI (Chang and Merzenich, 2003; de Villers-Sidani et al., 2007). Such behavior occurs because temporal and spectral selectivity of hearing is not ready at birth. Instead, the resolution of sound processing increases with age (de Villers-Sidani et al., 2007; Sanes and Bao, 2009). For instance, the width of inhibitory and excitatory receptive fields decrease and narrow with age, at the same time that frequency discrimination becomes more selective (Chang et al., 2005). Previous studies have also shown that abnormal development of the auditory cortex during the critical period for spectral tuning increases the number of broad receptive fields (Anomal et al., 2013). In this case, blockade of BDNF signaling during critical period can delay the maturation of inhibitory circuitry and impair the formation of the tonotopic map and single-peaked receptive fields (Anomal et al., 2013).

Considering the set of alterations in AI of VPA-treated rats observed in our study, it would be interesting to investigate whether these abnormalities could lead to disturbances of perception relevant enough to affect particular cognitive and emotional behaviors. In support to a significant impact on perception, Froemke et al. (2013) have recently shown that basal forebrain cholinergic modulation of rodent AI is able to reorganize cortical synapses and receptive field responses, and these changes are associated with performance improvements in an associative learning task.

### Inhibitory Circuitry in the Primary Auditory Cortex of VPA Animals

It is known that synaptic inhibition play an important role organizing and modulating receptive field responses both in auditory and visual cortices (Fangiolini and Hensch, 2000; Wang et al., 2002). In particular, parvalbumin-expressing interneurons, which comprise a large population (40%) of inhibitory cells with typical fast-spiking properties (Tamamaki et al., 2003), is directly implicated in the development and maintenance of receptive fields in the auditory cortical areas (del Rio et al., 1994; Sugiyama et al., 2008; Moore and Wehr, 2013). In addition, genetic models of autism and rats prenatally exposed to VPA have an unbalance of cortical inhibition that could explain some of their cortical processing dysfunctions and behavioral impairments (Gogolla et al., 2009). Therefore, we wondered if our VPA animals had a disruption in the inhibitory circuitry organization of AI.

Our results show that compared to controls, VPA-treated animals had similar densities of inhibitory parvalbumin-positive neurons in AI. Layer distribution analysis confirmed that these interneurons populate in equal numbers cortical layers II–VI of VPA and control rats. Therefore, the changes in receptive field responses and tonotopic map organization described in VPA rats here cannot be explained by quantitative differences in the organization parvalbumin-positive interneurons in AI. However, other mechanisms could account for such deficits. First, a distinct population of GABAergic interneurons could be under or overrepresented in AI producing the abnormal physiological responses of VPA rats (Ouellet and de Villers-Sidani, 2014). Second, it is possible that either excitatory or inhibitory disturbances at the synaptic level affect the balance between excitation and inhibition required for AI function. In fact, it has been shown that both excitatory and inhibitory dysfunctions in VPAs occur. Rinaldi et al. (2007) showed a reduction of NMDA receptors in the frontal cortex of VPA animals, whereas Banerjee et al. (2013) showed severe impairment of synaptic inhibition in the temporal cortex of VPAs. In the latter, the authors found a reduction in the frequency of miniature IPSCs, with slower kinetics, associated to deficits in presynaptic modulation.

In genetic models of autism, inhibitory circuitry is commonly affected. Mice that lack the homeodomain transcription factor Engrailed-2 (En-2) have reduced expression of parvalbumin and somatostatin in the hippocampus and cerebral cortex, but an augmented expression of parvalbumin-positive cells in lower layers of primary visual cortex (Sgadò et al., 2013). Interestingly, these animals have a normal development of acuity and visual receptive fields, though are deployed of experience-dependent plasticity during critical period (Allegra et al., 2014). Moreover, mutations in neuroligin-3 (Radyushkin et al., 2009), neuroligin-4 (Jamain et al., 2008), or neurexin-2 (Dachtler et al., 2014) produce autism-like phenotypes in mice with changes in the cortical inhibitory network, though not always with deficits in sensory processing which indicate that compensatory mechanisms and brain region specificities should be taken into account.

Together, our findings demonstrate that rats prenatally exposed to VPA display significant deficits in auditory processing, which might contribute to some of the behavioral disturbances observed in these animals, such as increased anxiety, abnormal fear responses and sensory hypersensitivity. It is plausible that the neurophysiological abnormalities observed in AI disclose a maturational dysfunction caused by the VPA interference during the early stages of brain development (E12.5), which leads to cortical synaptic dysfunctions at postnatal ages.

## Conflict of Interest Statement

The authors declare that the research was conducted in the absence of any commercial or financial relationships that could be construed as a potential conflict of interest.
