# Supplementary material for: Impaired Processing in the Primary Auditory Cortex of an Animal Model of Autism
Source: Front Syst Neurosci. 2015 Nov 16;9:158. doi: 10.3389/fnsys.2015.00158 (PMC4644803; doi:10.3389/fnsys.2015.00158)
Supplement: Supplementary file 1 [file Image_1.pdf]

Supplementary Figure 1 - VPA group

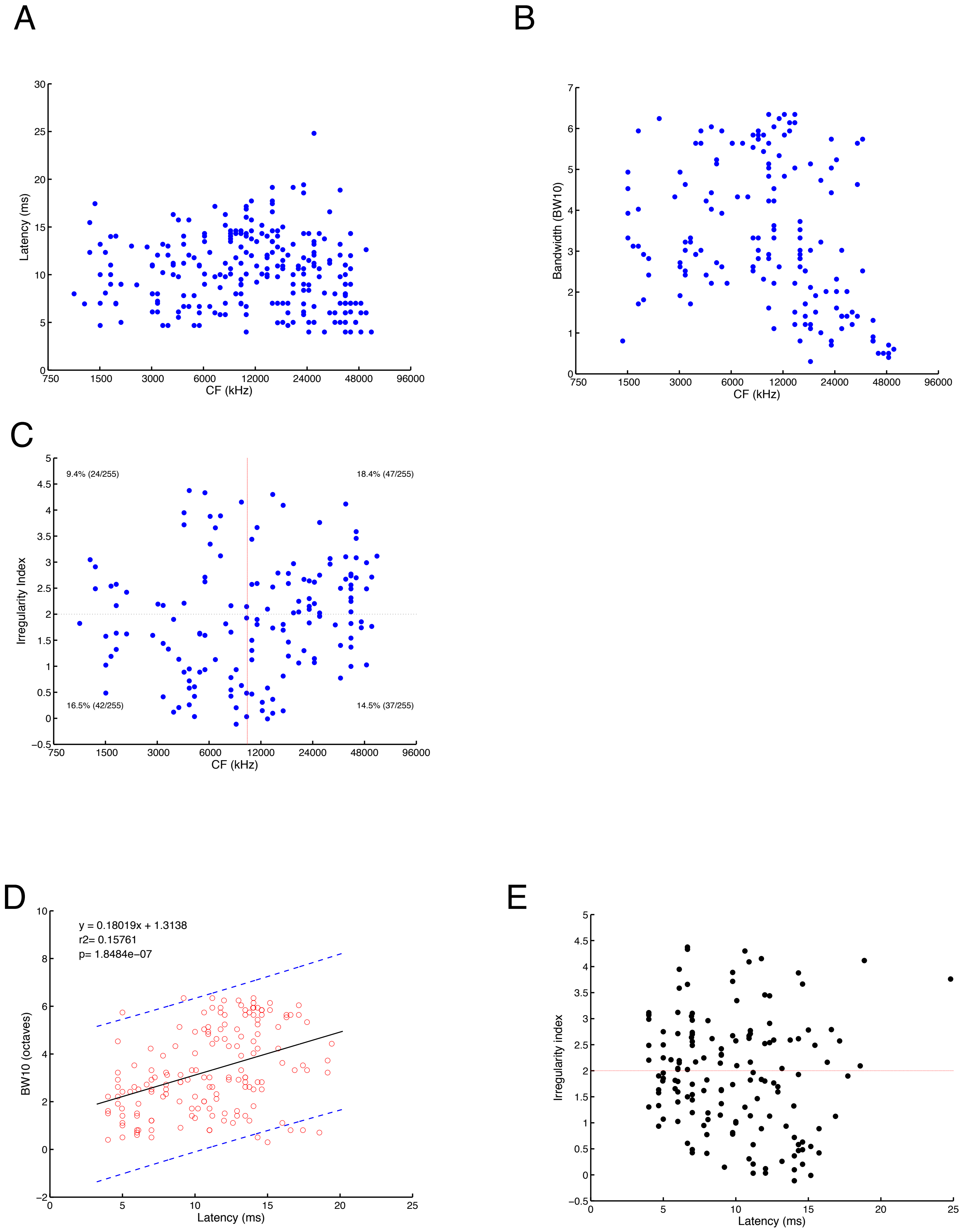

Supplementary Figure 1 - VPA group

F

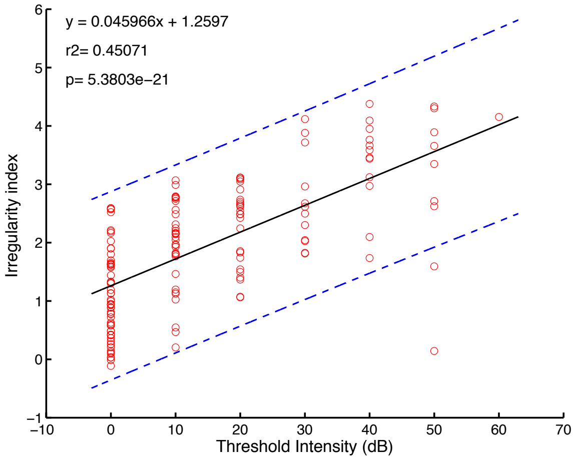

G

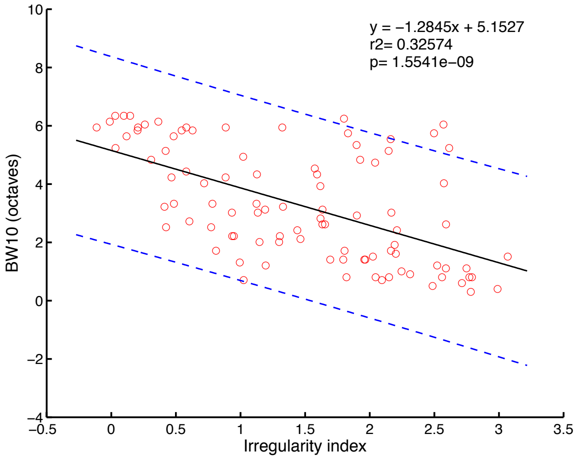

Figure S1: Pairwise correlation plots between combined receptive field parameters calculated from eight VPA animals. A total of 256 AI recording sites were used to reconstruct frequency-intensity receptive fields. Linear fit (black solid line) with 95% confidence interval (blue dashed line), coefficient of determination (r^2) calculated by Pearson's linear correlation and p-values are shown.
