# Supplementary material for: Impaired Processing in the Primary Auditory Cortex of an Animal Model of Autism
Source: Front Syst Neurosci. 2015 Nov 16;9:158. doi: 10.3389/fnsys.2015.00158 (PMC4644803; doi:10.3389/fnsys.2015.00158)
Supplement: Supplementary file 2 [file Image_2.pdf]

Supplementary Figure 2

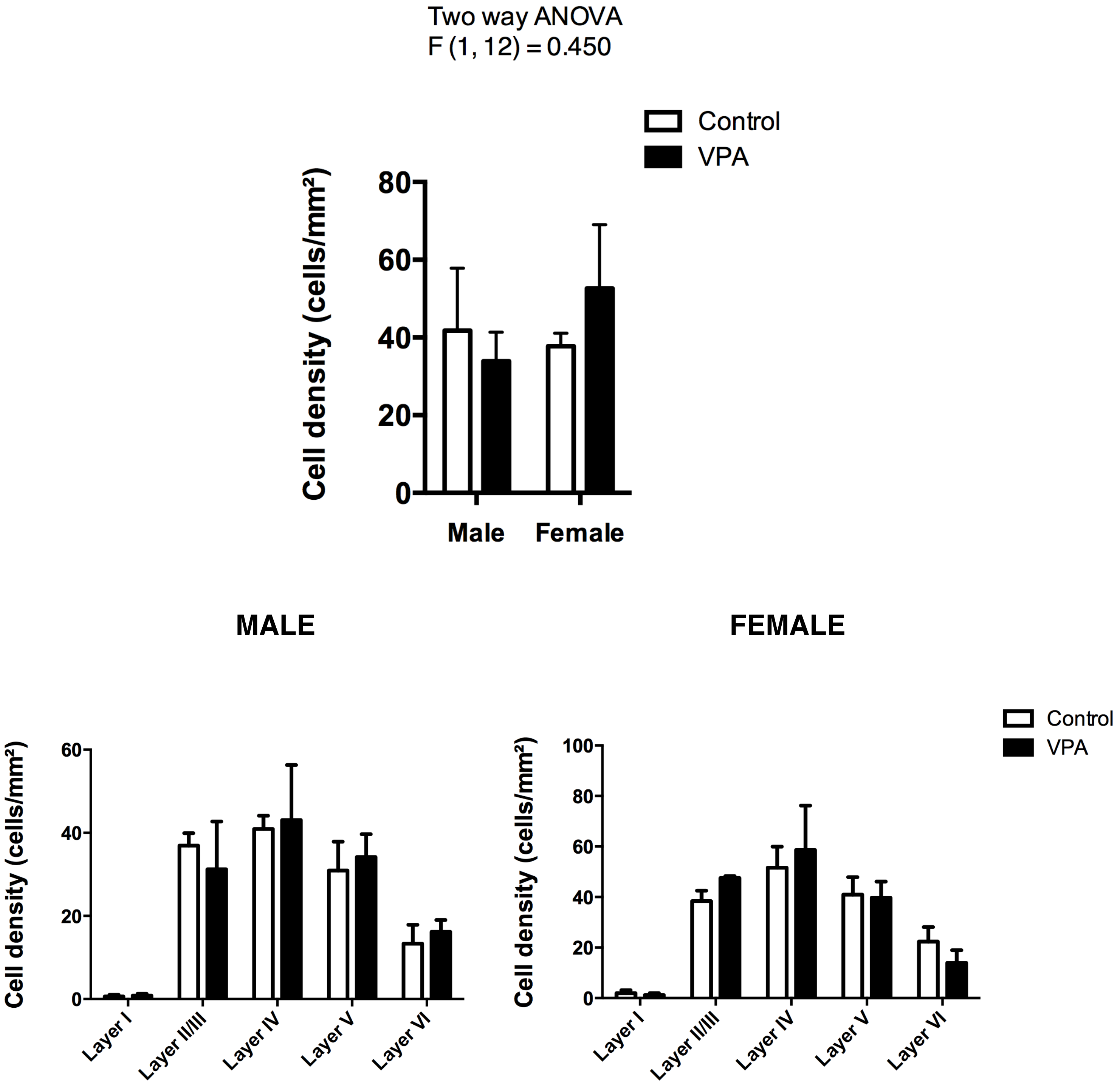

Figure S2: Parvalbumin-positive cell density in AI of male and female rats. No significant differences were observed between male and female animals from either Control and VPA group (two-way ANOVA, p>0.05). The statistical analysis across AI cortical layers also did not show any difference (two-way ANOVA, p>0.05). Control, males (N=4), females (N=5); VPA, males (N=4), females (N=3).
